# The impact of DSM classification changes on the prevalence of alcohol use disorder and ‘diagnostic orphans’ in Lebanese college youth: Implications for epidemiological research, health practice, and policy

**DOI:** 10.1371/journal.pone.0233657

**Published:** 2020-06-05

**Authors:** Lilian A. Ghandour, Sirine Anouti, Rima A. Afifi

**Affiliations:** 1 Department of Epidemiology and Population Health, Faculty of Health Sciences, American University of Beirut, Beirut, Lebanon; 2 Department of Community and Behavioral Health, College of Public Health, University of Iowa, Iowa City, Iowa, United States of America; 3 Department of Health Promotion and Community Health, Faculty of Health Sciences, American University of Beirut, Beirut, Lebanon; Radboud University, NETHERLANDS

## Abstract

**Background:**

Studies comparing prevalence of alcohol use disorder (AUD) using DSM-IV and DSM-5 diagnostic criteria in college students are limited. This study examines changes in AUD prevalence estimates using DSM-IV versus DSM-5 and characterizes the profile of DSM-5 “diagnostic orphans.”

**Methods and findings:**

A college student sample (n = 1,155; mean age: 21 ±1.97) selected conveniently from six large private and public universities in Greater Beirut, Lebanon completed an anonymous survey in May 2016. The study response rate was 83.1%. Data on DSM-IV and DSM-5 AUD criteria were gathered from 582 past-year drinkers, of which 377 (65%) were screened to have DSM-IV abuse/dependence, and 203 (35%) to have any DSM-5 AUD (58% mild, 21% moderate, and 21% severe). Overall percent agreement between measures was 68% (kappa = 0.41). One hundred and ninety-one students met one DSM-5 AUD criterion only (i.e. “diagnostic orphans,” herein DOs), of which the majority (82%) endorsed “hazardous use.” Compared to past-year drinkers with zero-endorsed DSM-5 criteria, DOs were more likely to be aged 21 or above [OR = 1.57(1.21–2.03)], less likely to perceive their socioeconomic status (SES) as poorer vs. same as others [OR = 0.17(0.07–0.43)], more likely to drink 1–2 times/week vs. ≤3 times per month [OR = 2.24(1.44–3.49)], and more likely to report past-year cigarette smoking [OR = 2.16(1.10–4.24)]. When compared to past-year drinkers with DSM-5 AUD, DOs were more likely to be pursuing a graduate or medical degree (vs. undergraduate degree) [2.06 (1.09–3.89)], and to be living with parents most of the time vs. not [OR = 2.68(1.14–6.31)]. DOs (versus drinkers with AUD) were less likely to drink at a high frequency (3–4 times /week or more vs.≤3 times per month) [OR = 0.15(0.05–0.48)], and to report past-year waterpipe smoking [OR = 0.54(0.34–0.85)], but more likely to report past-year marijuana use [1.89(1.10–3.23)]. The findings are subject to recall bias and under-reporting and the study could not infer causality because temporality of associations cannot be established in a cross-sectional study design.

**Conclusions:**

DSM-IV abuse/dependence prevalence rate was higher than DSM-5 AUD prevalence mainly due to the high percentage of students who engaged in “hazardous use”. The DO screen might capture a young person in transition between non-drinking/occasional drinking to drinking frequently/developing an AUD. The prevention, identification, and management of DOs may be critical components of a national alcohol harm-reduction policy.

## 1. Background

Alcohol Use Disorders (AUD) contribute significantly to the burden of mental health disorders [1]. Studies have also shown that individuals diagnosed with AUD commonly suffer from comorbid mental disorders [2–5], further adding to the overall burden. Disability-Adjusted Life Years (DALYs) attributed to alcohol have increased by more than 25% between 1990 and 2016, accounting for 4.2% and 5.2% of the total DALYs and deaths, respectively [6]. While AUD is more common in developed regions in the world, the potential harm is much higher in developing countries such as the Eastern Mediterranean region (EMR), where national alcohol harm reduction policies remain either absent or poorly enforced [7].

Alcohol drinking often begins in adolescence, as early as 13 years of age [8, 9]. Early initiation is particularly concerning because it has been linked to a higher likelihood of developing AUD later in life [10–12]. While rates of youth alcohol use seem to have stabilized in the U.S. [13, 14], rates continue to rise in developing countries [9]. This increase in alcohol use and its associated harms are closely linked to the globalization of economic markets with transnational alcohol companies emerging in poorer countries [15, 16].

For almost two decades (1993–2014), the *Diagnostic and Statistical Manual of Mental Disorders* 4^th^ Edition (DSM-IV) [17, 18] had helped clinicians to classify and screen for alcohol abuse and alcohol dependence and had helped researchers systematically screen for alcohol-related problems in the community [19–23]. The release of DSM-5 in 2013 brought some important changes to AUD terminology and classification criteria. Mainly, the diagnosis shifted from dichotomized measures of alcohol abuse (one out of four symptom criteria required) and alcohol dependence (three out of seven symptom criteria required) in DSM-IV, to a spectrum of DSM-5 AUD ranging from mild (2 to 3 out of eleven criteria endorsed) to severe (6 or more criteria); presence of two or more criteria indicates presence of any DSM-5 AUD (Table 1) [17, 18]. Changes were also made to the criteria: “legal problems due to alcohol use” was dropped, and “craving” was added [17, 18]. In a recent systematic review, the authors concluded that DSM-5 generally inflates the AUD prevalence when compared to DSM-IV [24], although some studies did report a lower DSM-5 AUD prevalence rate compared to the combined DSM-IV alcohol abuse/dependence rate [25, 26]. This evidence is largely based on adult surveys [24], with only three studies focused on college students or youth, which concluded that AUD prevalence seem to be higher using DSM-5 diagnostic criteria [27–29].

**Table 1:** Criteria used to screen for DSM-IV abuse/dependence and DSM-5 AUD.

Criteria	DSM-IV Abuse^a^	DSM-IV Dependence^b^	DSM-5 AUD^c^
1. Recurrent drinking resulting in failure to fulfill role obligations	**✔**	**−**	**✔**
2. Recurrent drinking resulting in hazardous situations (It was sufficient to meet this criterion if either of the following was reported):	**✔**	**−**	**✔**
a) on more than one occasion operated a car or other vehicle shortly after having several drinks of alcohol, or			
b) found themselves more than once in a situation that increased their chances of getting injured after having had too much alcohol			
3. Continued alcohol drinking despite alcohol-related social or interpersonal problems	**✔**	**−**	**✔**
4. Recurrent alcohol-related legal problems	**✔**	**−**	**✕**
5. Tolerance	**−**	**✔**	**✔**
6. Withdrawal or substance use for relief/avoidance of withdrawal	**−**	**✔**	**✔**
7. Drinking in larger amounts or for longer than intended	**−**	**✔**	**✔**
8. Persistent desire/unsuccessful attempts to stop or reduce drinking	**−**	**✔**	**✔**
9. Great deal of time spent obtaining, using, or recovering from alcohol	**−**	**✔**	**✔**
10. Important activities given up/reduced because of drinking	**−**	**✔**	**✔**
11. Continued drinking despite knowledge of physical or psychological problems caused by alcohol	**−**	**✔**	**✔**
12. Alcohol craving	**✕**	**✕**	**✔**

^a^ DSM-IV Abuse: any one or more abuse criteria within the past 12 months and no dependence diagnosis

^b^ DSM-IV Dependence: any three or more dependence criteria within the past 12 months

^c^ DSM-5 AUD: Any two or more AUD criteria within the past 12 months, further grouped into Mild (2–3), Moderate (4–5), and Severe (6+) AUD

In addition to generating inconsistent prevalence estimates, the change from DSM-IV to DSM-5 has not fully addressed the issue of “diagnostic orphans.” The term ‘diagnostic orphans’ (DO) was coined by Kaczynski & Martin (1995) to describe a group of individuals with one or two DSM-IV alcohol dependence criteria and no alcohol abuse criteria, thus receiving no formal diagnosis [30–32]. DOs have therefore constituted a hidden and overlooked group of problem alcohol drinkers (i.e. drinkers with health or social problems related to their alcohol use) who are at a high risk of progressing to AUD [30]. In a large cross-national community study of the World Mental Health Surveys in nine countries, of which Iraq was the only Arab country, DSM-IV DOs made up 5.3% of the total sample of 44,341 respondents aged between 18 and 97 years [26]. Among young people, previous studies have shown considerable proportions of DSM-IV DOs ranging between 10% and 30% [28, 32–35].

By re-classifying some of the DSM-IV DOs as having “mild AUD,” the DSM-5 revisions partially improve detection of problem alcohol drinkers. Yet, individuals who fulfill only one of the eleven DSM-5 criteria continue being undetected and thus can be labeled as ‘DOs' [30]. This subgroup may still include problem drinkers who, despite not fulfilling DSM criteria for an AUD [30], may eventually inflict harm on themselves and/or others, if left undetected [35]. In the U.S., DSM-5 criteria DOs were shown to consume more alcohol and have more substance-related problems than those who met none of the DSM-5 criteria [28].

A clear research gap still exists in understanding how DSM changes have affected AUD prevalence in young people outside of the Americas, and in understanding the implications of these changes on measuring DOs specifically. AUD among college youth is of particular public health concern since it can have detrimental effects, including poor academic performance and dropout, unintentional injuries, health problems, heightened risk of engaging in other risky practices, legal problems, increased risk of other substance use disorders in adulthood, and death [36]. Therefore, accurate screening of AUD in college samples is critical for more adequate planning and implementation of early, evidence-based interventions to prevent morbidity and mortality [37].

Using a college sample from Greater Beirut, Lebanon, this study aims: (1) to compare the past-year DSM-IV and DSM-5 prevalence in a college sample of past-year drinkers; and (2) to examine the extent of DOs, and to compare their substance use profiles (i) to drinkers with no DSM-5 AUD criteria endorsed and (ii) to drinkers with 2 or more AUD criteria endorsed (i.e. having any AUD). The findings will add to the limited literature on this topic among college youth and address the gap on this issue within the developing world.

## 2. Methods

### 2.1 Study design and population

University students (n = 1,155) were conveniently sampled from six large private and public university campuses in Greater Beirut, Lebanon in May 2016. Universities were purposively selected to ensure that the sample included students of diverse backgrounds; including their socioeconomic status, using tuition fees as proxy (for an engineering degree, for example, the credit cost ranged from USD 66/credit to USD 824/credit across the sampled universities); and their educational background (using primary language of instruction as proxy including Arabic, French, or English). Trained data collectors approached the students off campus. Only students aged 18 years or older and enrolled in one of the targeted universities were included in the study. After obtaining oral consent, the students were asked to complete an anonymous survey using paper and pencil, either in English or Arabic based on their preference. Ethical approval was obtained from the Institutional Review Board (IRB) of the American University of Beirut. The study response rate was 83.1%.

The present study focuses on the 582 students that reported drinking alcohol within the year preceding the survey (i.e. past-year drinkers). AUD using both DSM-IV and DSM-5 criteria were assessed among this subsample of university student respondents.

### 2.2 Measures

The survey was initially developed by our team in English, then translated into Arabic, and back translated into English. It included questions on socio demographics (e.g., age, gender, nationality, current degree, living arrangement); on alcohol consumption patterns (e.g., past-year use, frequency of past-year use of different beverage types, methods of obtaining alcohol, alcohol-related problems based on DSM-5 criteria); on alcohol control policies (e.g., students’ perception of policies and their impact on alcohol consumption), and on other health-related risk behaviors (e.g., use of tobacco, use of illegal drugs, and non-medical use of psychoactive prescription medications). The overarching theoretical frameworks that guided the development of the questionnaire included: (1) the World Health Organization’s set of evidence-based “best buy” core intervention areas for non-communicable disease (NCD) risk factors (i.e. affordability, availability, advertising/marketing, and drink driving) that have been shown to influence alcohol consumption behaviors [38], and (2) the International Tobacco Control (ITC) study framework [39]. Questions were also borrowed from various other studies including national and international surveys such as the National Survey on Drug Use and Health (NSDUH), the National Epidemiologic Survey on Alcohol and Related Conditions(NESARC), Gender, alcohol and culture: an international study (GENACIS), and International Alcohol Control (IAC) study [40–42], among others. The pre-final version of the questionnaire was pre-tested by the trained fieldworkers with six students to ensure all the questions were clear and comprehensible. The data used for this study are part of a larger research initiative aimed at gathering evidence to inform a national alcohol policy for Lebanon.

#### 2.2.1 Alcohol-use disorder (AUD)

Thirteen questions about alcohol-related problems mapped onto 12 criteria that allowed us to generate both a DSM-IV and DSM-5 diagnosis of past-year AUD (Table 1). We used the questionnaire published in Mohler-Kuo et al. (2015), which is based on the Semi-Structured Assessment for the Genetics of Alcoholism (SSAGA) as adapted by Knight et al. (2002) [43]. In our study, the Kuder-Richardson (KR-20) coefficient of internal consistency reliability was 0.82. Using these questions and the DSM-IV and DSM-5 criteria, three binary (yes/no) variables and one categorical variable were created. ‘DSM-IV abuse but no dependence” was defined as having ≥1 abuse criterion and no dependence criteria endorsed. ‘DSM-IV abuse or dependence’ was defined as having ≥1 abuse criterion or ≥ 3 dependence criteria endorsed. Any ‘DSM-5 AUD’ was defined as having ≥ 2 of the 11 DSM-5 AUD criteria endorsed (i.e. AUD positive); ‘DSM-5 AUD severity’ further classified those with any DSM-5 AUD as mild (i.e. 2–3 criteria), moderate (i.e. 4–5 criteria), or severe (i.e. 6+ criteria) [44, 45].

#### Diagnostic orphans (DOs)

Two additional variables were created to measure “diagnostic orphans” (DOs), to indicate past-year drinkers who endorsed only one or two of the alcohol dependence criteria and no abuse criteria (in the case of DSM-IV classification), or endorsed only one criterion of the 11 criteria (in the case of DSM-5 classification).

#### 2.2.3 Alcohol and other substance use

The analysis in this study was restricted to past-year alcohol drinkers (i.e. students who reported having had more than just a few sips of an alcoholic drink in the year prior to the survey). The assessment of alcohol consumption patterns and use of other substances was restricted to the past year (i.e. the 12 months preceding the survey). Past-year frequency of alcohol consumption was reported to be either 1–3 times/month or less, 1–2 times/week, or 3–4 times/week or more. Binary questions (yes/no) measured use of other substances. Past-year cigarette and waterpipe smoking were measured separately by asking students if they had smoked one or more cigarettes per day, and had one or more waterpipe smoking sessions, at least once per week. Any past-year use of other substances was also assessed: (a) past-year marijuana or hashish use; (b) past-year other illegal drug use such as cocaine, heroin, stimulants (methamphetamines, Captagon), hallucinogens, or party drugs (ecstasy); and (c) past-year nonmedical use of psychoactive prescription drugs (e.g., use of Xanax, Tramal, Valium or others without a doctor’s advice).

#### 2.2.4 Demographic information

Demographic data that was assessed included: gender (male/female), age (in years), university/college degree being pursued (Bachelor’s/Master’s/Medical/Doctoral degree), place where they live most of the time (alone or with a roommate; with parents or relatives; in a single or mixed sex dormitory), and self-perceived socioeconomic status (SES) (richer than; about the same as; poorer than most other young people their age).

### 2.3 Statistical analysis

Data analyses were performed using Stata version 13 [46]. Prevalence rates of past-year DSM-IV and DSM-5 AUD were compared using relative frequencies. Overall percent agreement and Cohen’s Kappa were computed to explore agreement between the DSM-IV and DSM-5AUD classifications of prevalence estimates. We employed Pearson’s Chi-square test to compare characteristics of past-year drinkers in the three mutually exclusive DSM-5 AUD groups (AUD = 0, AUD = 1, and AUD = 2+). We then ran a multivariable regression model with all correlates that had a *p*-value<0.05 at the bivariate level in Table 4. Statistical significance was set at alpha<0.05.

## 3. Results

### 3.1 Sample characteristics

Past-year drinkers consisted of a slightly higher proportion of males (58%) than females (42%). About two-thirds (64%) were aged more than 21 years, 75% were living with their parents, and 77% were working towards their Bachelors of Science (BS) degree. As for students’ perceptions of their socioeconomic status, 45% perceived themselves as being “as well off as others their age,” another 52% perceived themselves as being richer than others, and the remaining (3%) perceived themselves as poorer. All past-year drinkers were either Lebanese (97%) or dual nationals (3%), holding both a Lebanese and another nationality; hence, the analyses and results are constricted to Lebanese nationals.

### 3.2 AUD prevalence estimates as per DSM- IV and DSM-5 criteria

The 13 questions (mapped onto the 12 AUD criteria) are presented in Table 1. Among past-year drinkers, 62% (n = 329) were screened to have DSM-IV alcohol abuse (≥1 criteria), and 65% (n = 377) to have alcohol abuse or dependence. Using DSM-5 criteria, 35% (n = 203) of the past-year drinkers were screened to have any AUD: 20% had mild AUD, 7% moderate AUD and 7% severe AUD. Overall percent agreement was 68% (kappa = 0.41) indicating only a fair agreement between DSM-IV alcohol abuse/dependence and DSM-5 AUD.

Table 2 presents the results of a cross-tabulation between DSM-IV and DSM-5 categories of AUD; the percentage of students who fulfilled DSM-IV categories of AUD (no abuse/no dependence, abuse/no dependence; abuse or dependence) within DSM-5 AUD categories are described. We note that of the 203 students who scored positive on DSM-5 AUD, the overwhelming majority (97.04%) also screened positive for DSM-IV abuse or dependence. Within the 379 past-year drinkers who were screened negative on DSM-5 defined AUD, almost half (47%) were screened positive for DSM-IV abuse or dependence (driven by the abuse classification) (Table 2- Part A). Past-year drinkers with no DSM-5 AUD may have endorsed zero or one DSM-5 criteria for AUD. As shown in Table 2 (Part B), among those with zero-endorsed DSM-5 criteria, about 99% also did not fulfill DSM-IV-defined abuse or dependence; the exception was two students that endorsed the DSM-IV legal problems criterion, which is no longer a criterion in DSM-5. However, of the 191 alcohol-drinking students who endorsed only one of the eleven DSM-5 AUD criteria (i.e. DSM-5 DOs), 93% (n = 178) screened positive for DSM-IV abuse or dependence (driven by alcohol abuse).

**Table 2:** Comparing estimates of DSM-IV abuse/dependence and DSM-5 AUD among 582 past-year alcohol drinking university students from Greater Beirut, Lebanon.

		DSM-IV classification		
		DSM-IV Abuse or dependence		DSM-IV Abuse only (no dependence)
		No	Yes	Yes
	DSM-5 Classification	%(n)	%(n)	%(n)
**Part A**	**No AUD** *(n = 379)*	52.51 (199)	47.49 (180)	47.49 (180)
	**Any DSM-5 AUD positive** *(n = 203)*	2.96 (6)	97.04 (197)	73.40 (149)
**Part B**	**0 DSM-5 criteria** *(n = 188)*	98.94 (186)	1.06 (2)	1.06 (2)
	**1 DSM-5 criterion** *(n = 191)*	6.81 (13)	93.19 (178)	93.19 (178)
	**2–3 DSM-5 criteria (mild)** *(n = 118)*	5.08 (6)	94.92 (112)	94.92 (112)
	**4–5 DSM-5 criteria (moderate)** *(n = 42)*	0	100 (42)	100 (35)
	**6+ DSM-5 criteria (severe)***(n = 43)*	0	100 (43)	100 (2)

### 3.3 Characteristics of the DSM-5 Diagnostic Orphans (DOs) using DSM-5 criteria

Table 3 describes the distribution of each of the 11 DSM-5 AUD criteria in the total sample of past-year drinkers and within subcategories of DSM-5 AUD: zero-endorsed DSM-5 criteria (AUD = 0), DOs with only one DSM-5 criterion endorsed (i.e. AUD = 1), and past-year drinkers who screened positive for DSM-5 AUD (i.e. AUD = 2+). Focusing on the DOs (i.e. AUD = 1), we note that the most commonly reported DSM-5 AUD criterion was ‘physically hazardous alcohol use’ (91.62%, n = 175), which reflects “recurrent alcohol use in situations in which it is physically hazardous even though actual harm might not have occurred.” Only five (2.62%) of DSM-5 DOs met the DSM-IV criterion of ‘legal problems’, which was dropped in DSM-5.

**Table 3:** Distribution of DSM-5 AUD criteria in the total sample of 582 past-year alcohol drinkers from Greater Beirut, Lebanon, and within subcategories of DSM-5 defined AUD.

		No DSM-5 AUD		Any DSM-5 AUD
	Overall past-year drinkers % (n)	AUD = 0▾ % (n)	AUD = 1▾% (n)	AUD = 2+▾ % (n)
Unable to fill role obligations	25.09 (146)	0	0	71.92 (146)
Consume in physically hazardous situations*	51.20 (298)	0	91.62 (175)	60.59 (123)
Recurrent alcohol-related legal problems *(not included in DSM-5)*	6.19 (36)	1.06 (2)	2.62 (5)	14.29 (29)
Social/interpersonal problems	13.40 (78)	0	1.57 (3)	36.95 (75)
Tolerance	18.38 (107)	0	4.19 (8)	48.77 (99)
Withdrawal	7.39 (43)	0	0	21.18 (43)
Larger amounts/longer than intended	3.78 (22)	0	0	10.84 (22)
Inability to cut down	1.55 (9)	0	0	4.43 (9)
Great deal of time spent in activities obtaining alcohol	3.78 (22)	0	0	10.84 (22)
Important activities given up	25.09 (146)	0	0	71.92 (146)
Recurrent physical/psychological problems	9.28 (54)	0	0	26.60 (54)
Craving	13.92 (81)	0	2.62 (5)	37.44 (76)

*(i.e. on more than one occasion operated a car or other vehicle shortly after having several drinks of alcohol, or found themselves more than once in a situation that increased their chances of getting injured after having had too much alcohol)

▾AUD = 0 means student endorsed no DSM-5 criteria; AUD = 1 means student endorsed only 1 DSM-5 criterion; AUD 2+ means student endorsed two or more DSM-5 criteria

Table 4 compares the socio-demographic and substance use characteristics of drinkers with only one DSM-5 criterion endorsed (AUD = 1) against those of drinkers with zero-endorsed DSM-5 criteria (AUD = 0) and two or more endorsed DSM-5 criteria (AUD = 2+). Compared to past-year drinkers with zero-endorsed DSM-5 AUD criteria, DOs were more likely to be males, aged 21 years or above, and less likely to perceive their SES as poorer than vs. the same as others. DOs were also more likely to report drinking alcohol 1–2 times/week rather than 1–3 times/month or less, and to report past-year cigarette smoking, compared to past-year drinkers with zero-endorsed DSM-5 criteria. Compared to past-year drinkers with any DSM-5 AUD (i.e. AUD = 2+), DOs were more likely to be aged 21 years or above, and to report living with their parents for most of the time. DSM-5 DOs, as compared to those with any DSM-5 AUD, were less likely to drink frequently (3–4 times per week or more vs. 1–3 times per month or less) and to report waterpipe smoking (Table 4).

**Table 4:** Distribution of DSM-5 AUD and AUD categories by socio-demographic and substance use characteristics of 582 past-year alcohol drinking university students from Greater Beirut, Lebanon.

	Bivariate analysis					Multivariable regression analysis^ᴪ^	
	AUD = 0%(n)	AUD = 1%(n)	AUD = 2+ %(n)	P-value AUD = 1 vs. AUD = 0 (reference)	P-value AUD = 1 vs. AUD = 2+ (reference)	AUD = 1 vs. AUD = 0 OR (95% CI)	AUD = 1 vs. AUD = 2+ OR (95% CI)
***Gender***							
Males	50.53 (95)	60.73 (116)	62.56 (127)	0.046*	0.709	1.51 (0.99–2.32)	0.97 (0.64–1.48)
Females (ref)	49.47 (93)	39.27 (75)	37.44 (76)			1.00	1.00
***Age***							
<21 years (ref)	38.30 (72)	26.70 (51)	42.36 (86)	0.016*	0.001*	1.00	1.00
21 years or more	61.70 (116)	73.30 (140)	57.64 (117)			1.57 (1.21–2.03)*	1.63 (0.93–2.88)
***Pursuing degree***							
Bachelor degree (ref)	73.40 (138)	74.35 (142)	83.25 (169)	0.835	0.030*	1.00	1.00
Medical or graduate degree	26.60 (50)	25.65 (49)	16.75 (34)			0.73 (0.47–1.14)	2.06 (1.09–3.89)*
***Living with parents most of the time***							
Yes	78.72 (148)	83.77 (160)	63.05 (128)	0.208	<0.001*	1.29 (0.47–3.55)	2.68 (1.14–6.31)*
No (ref)	21.28 (40)	16.23 (31)	36.95 (75)			1.00	1.00
***Perceived SES***							
Same as others (ref)	37.77 (71)	48.69 (93)	48.28 (98)	0.012*	0.994	1.00	1.00
Poorer than others	5.32 (10)	1.05 (2)	0.99 (2)			0.17 (0.07–0.43)*	1.53 (0.16–14.0)
Richer than others	56.91 (107)	50.26 (96)	50.74 (103)			0.53 (0.21–1.36)	1.42 (0.86–2.34)
***Frequency of drinking***							
1–3 times/month or less (ref)	57.45 (108)	43.98 (84)	25.12 (51)	<0.001*	<0.001*	1.00	1.00
1–2 times /week	21.81 (41)	45.55 (87)	33.99 (69)			2.24 (1.44–3.49)*	0.73 (0.41–1.33)
3–4 times /week to daily or nearly everyday	20.74 (39)	10.47 (20)	40.89 (83)			0.53 (0.21–1.36)	0.15 (0.05–0.48)*
***Past-year cigarette smoking***							
Yes	43.62 (82)	66.49 (127)	79.89 (150)	<0.001*	0.108	2.16 (1.10–4.24)*	0.80 (0.37–1.71)
No (ref)	56.36 (106)	33.51 (64)	26.11 (53)			1.00	1.00
***Past-year waterpipe smoking***							
Yes	45.21 (85)	35.08 (67)	58.13 (118)	0.044*	<0.001*	0.79 (0.47–1.35)	0.54 (0.34–0.85)*
No (ref)	54.79 (103)	64.92 (124)	41.87 (85)			1.00	1.00
***Past-year marijuana use***							
Yes	6.91 (13)	16.23 (31)	13.30 (27)	0.005*	0.412	2.26 (0.94–5.44)	1.89 (1.10–3.23)*
No (ref)	93.09 (175)	83.77 (160)	86.70 (176)			1.00	1.00
***Past-year other illegal drug use***							
Yes	3.72 (7)	4.19 (8)	5.42 (2)	0.816	0.569	-	-
No (ref)	96.28 (181)	95.81 (183)	94.58 (192)				
***Past-year use of non-prescribed prescription drugs***							
Yes	2.66 (5)	2.62 (5)	6.40 (13)	0.980	0.072	-	-
No (ref)	97.34 (183)	97.38 (186)	93.60 (190				

* P-value <0.05 using Pearson’s Chi-square test; (ref): reference category

^**ᴪ**^ Controlling for all correlates that had a P-value<0.05 at the bivariate level (i.e. gender, age, pursuing degree, living with parents most of the time, perceived SES, frequency of drinking, past-year cigarette smoking, past-year waterpipe smoking, and past-year marijuana use)

Multivariable regression analyses (Table 4) included all correlates that had a p-value < 0.05 at the bivariate level (i.e. gender, age, pursuing degree, living with parents most of the time, perceived SES, frequency of drinking, past-year cigarette smoking, past-year waterpipe smoking, and past-year marijuana use). Specifically, DOs (compared to past-year drinkers with zero-endorsed DSM-5 AUD criteria) were more likely to be aged 21 years or above vs. <21 [OR = 1.57(1.21–2.03)] and less likely to perceive their SES as poorer vs. same as others [OR = 0.17(0.07–0.43)]. Quite importantly, they were also more likely to drink alcohol 1–2 times/week vs. less frequently (≤3 times per month) [OR = 2.24(1.44–3.49)], and more likely to report past-year cigarette smoking [OR = 2.16(1.10–4.24)]. Compared to past-year drinkers with DSM-5 AUD, DOs were more likely to be pursuing a graduate or medical degree vs. undergraduate degree [2.06 (1.09–3.89)] more likely to be living with parents most of the time [OR = 2.68(1.14–6.31)], less likely to drink at a high frequency (3–4 times /week or more) than low (≤3 times per month) [OR = 0.15(0.05–0.48)], and less likely to report past-year waterpipe smoking [OR = 0.54(0.34–0.85)] but more likely to report past-year marijuana use [1.89(1.10–3.23)].

## 4. Discussion

In this college student sample of past-year drinkers from Lebanon, almost twice as many students met DSM-IV abuse or dependence criteria than DSM-5 AUD criteria (65% vs. 35%, respectively). This fair/moderate agreement (kappa = 0.41) was lower than that reported in other populations: 0.6 in college student samples [31], between 0.79 and 0.90 in clinical/institutional samples, and between 0.50 and 0.85 in non-clinical samples [24]. At face value, our findings do not corroborate recent conclusions of a systematic review that DSM-5 diagnostic criteria inflate prevalence rates of AUD compared with DSM-IV [24]. In the review, the authors explained the DSM-5 inflated estimates mainly by the higher number of DSM-IV ‘diagnostic orphans’ (i.e. 1 or 2 DSM-IV dependence criteria) compared to DSM-IV single-criterion alcohol abuse individuals [24]. In our study, the scenario was the opposite. Results showed a higher percentage of drinkers who met DSM-IV criteria for abuse only (62%) than those who screened positive for 1 or 2 DSM-IV dependence criteria only (i.e. DOs), which explains the inflated percentage of DSM-IV abuse or dependence in our study.

The percentage of DSM-5 DOs in our sample [mean age 21.36 ± 1.83 years] of past-year drinkers (33%) was slightly higher than that found (24%) in a U.S.- based college sample [mean age 20.11 ± 1.79 years] [28]. Compared to those with zero-endorsed DSM-5 criteria, DSM-5 DOs were more likely to report past-year daily cigarette use, any past-year marijuana use, and to drink alcohol more frequently. This finding is consistent with Hagman and colleagues’ representation of DOs as a ‘distinct group of drinkers’ who engage in risky levels of alcohol and other drug use [28], and also supports published literature on possible co-occurrence of risk [47, 48].

The discordance in estimates between DSM-IV and DSM-5 criteria in this college sample was found to be due to the high percentage of DSM-IV alcohol abuse, driven namely by the hazardous alcohol use sub-criterion: driving while impaired (i.e. ‘*operated a car or other vehicle shortly after you had had several drinks of alcohol’*). Thus, youth whose only alcohol-related problem is driving under alcohol influence would fulfil DSM-IV alcohol abuse criteria, but not meet the threshold for any DSM-5 AUD. Using DSM-5 AUD classification may therefore result in a “missed opportunity” to detect drinkers with a potentially serious alcohol-related problem. The extent of reported hazardous alcohol use has been linked to the level of enforcement of drink driving laws [49–51]. Therefore, this “missed opportunity” may be more common in countries such as Lebanon, where drink-driving regulations are loosely enforced [7]. Critiques of the DSM-IV drink driving criterion suggest it tends to potentially over-diagnose AUD; in one study, nearly 70% of surveyed adults met criteria for alcohol abuse solely on the basis of the drink-driving variable [52]. Indeed, further analyses of our data confirmed that the DSM-IV abuse/dependence prevalence would be reduced by half had those who scored positive on ‘hazardous use’ actually scored negative. Some researchers have questioned the validity of the AUD diagnosis that relies on the ‘hazardous use’ indicator given its high prevalence rate [50, 53]. Whether it is truly that prevalent or a methodological artifact caused by age-related misinterpretation of classification criteria has also been investigated, and authors have shown that the hazardous use (and tolerance criteria) are communicated adequately and understood as intended by young adults [54]. While Slade at al. (2013) demonstrated that young adults do understand and adequately interpret the interview questions designed to operationalize the hazardous use criterion, there still remains ongoing debate regarding the validity and utility of this criterion, and some researchers have proposed that this criterion be removed from the DSM-5 diagnostic criteria for AUD [49, 51]. Nonetheless, others have suggested that the criterion could reflect a psychiatric disorder rather than a sociocultural factor, and thus should be considered as an appropriate diagnostic criterion [55].

The findings of the present study should be interpreted in light of some limitations. The self-reported data may be subject to recall bias or under-reporting, since alcohol consumption in the Middle Eastern region is considered a sensitive topic; still, the anonymous nature of the survey is likely to have reduced such information bias. The prevalence estimates of DSM-IV or DSM-5 AUD results, or the profile of the DOs, may not be generalizable to young drinkers in other universities or even non-college youth drinkers given the convenient nature of the sampling. Still, the internal validity of the results is likely not to be affected given that our primary objective relied on comparing the responses of the same students on various criteria, and selection bias is unlikely to affect the comparative validity of student responses. Post-hoc adjustments for multiple comparisons were also not conducted as we believed that the process oversimplifies a complex issue in hypothesis testing; also, in the case of exploratory studies with clear pre-planned hypotheses, a strict adjustment for multiple comparisons is less critical [56].

Overall, this study adds to the scant literature comparing DSM-IV versus DSM-5 diagnoses of AUD among young college drinkers and is, to our knowledge, the only research evidence to date on youth from a developing country. The study illustrates the potential for high agreement between the two criteria (DSM-IV and DSM-5) if it were not for the ‘hazardous alcohol use’ criterion, which may be more commonly reported in contexts where drink driving is an issue due to lax laws and enforcement. Unlike in DSM-IV, where a young person can screen positive for alcohol abuse by endorsing only one alcohol-related problem (e.g., hazardous use), the DSM-5 AUD diagnosis based on the endorsement of two or more criteria could miss detecting drinkers with a DUI (drinking under the influence) problem. While DUI alone is not necessarily an indication of an AUD, it can be a warning sign of preventable harm to oneself and to others. One concern is that DSM-5 AUD classification might miss young people in high-risk groups, such as repeat DUI offenders, that DSM-IV alcohol abuse would have captured, which may result in fewer people receiving any attention or care for their recidivism. Targeted educational programs or other community prevention programs may not suffice for this group of recidivists [57]. While DOs would need early detection and management, population-based and evidence-informed public health prevention measures and adequately enforced legislations such as tighter drink-driving regulations remain key for preventing DUI [58, 59], and reducing the risk of “over-pathologizing” human behaviors [60]. From a population-based epidemiological perspective where the identification of high-risk groups does not necessarily mean their clinical management, our findings on the profile of DOs highlight the importance of primary and secondary preventive programs and the effective enforcement of drink-driving laws.

Separate from the important methodological analysis, the high prevalence of AUD among our convenient sample of young college drinkers from Greater Beirut, Lebanon is concerning especially coupled with our knowledge of the high alcohol outlet density in areas surrounding schools and universities [61]. The assessment of DSM-5 AUD in college studies is not common. The majority of studies have employed the Alcohol Use Disorders Identification Test (AUDIT), a screening tool that has detected prevalence estimates as high as 66.4% in Ireland, 68.9% in the US, or as low as 27.5% and 6.9% respectively among male and female college students in Tanzania [62, 63]. Only two studies have assessed DSM-5 AUD among college samples and reported lower past year prevalence estimates of 27.7% and 50% in the southeastern and northeastern parts of the U.S., respectively [28, 31].

## 5. Conclusion

Early detection of problem drinking among college students is a critical component of any national alcohol harm-reduction strategy. In a country like Lebanon, where laws governing DUI are loosely enforced, DSM-IV alcohol abuse/dependence may be higher than DSM-5 AUD prevalence driven by the high endorsement of the ‘hazardous alcohol use’ criterion. Still, the latter may provide a more nuanced picture of potential harm from alcohol use among young persons in countries where alcohol policies are loosely enforced. Young people screened as DOs may have been assessed during their journey from non-drinking/occasional drinking to drinking frequently/developing an AUD. Indeed, the DSM-5 DOs in our sample, the majority of whom had reported driving while impaired, were more likely to use other substances compared to drinkers with zero-endorsed DSM-5 criteria, and less likely compared to drinkers with any DSM-5 AUD. More generally, a national alcohol-harm reduction strategy that includes proper screening and evidence-informed primary/secondary prevention efforts is critically needed to protect young persons from alcohol-related harms and to ensure that additional alcohol-related problems do not develop among DOs.
